# In Vitro Antifungal Activity of Essential Oils and Nanoemulsions of *Zingiber cassumunar* and *Cymbopogon citratus* Against Planktonic and Biofilm Forms of *Malassezia pachydermatis*

**DOI:** 10.3390/antibiotics15040402

**Published:** 2026-04-16

**Authors:** Sirikorn Promcham, Orawan Limsivilai, Theerawat Kritsadasima, Suttiwee Chermprapai, Natthasit Tansakul, Pareeya Udomkusonsri, Chompoonek Yurayart

**Affiliations:** 1 Department of Microbiology and Immunology, Faculty of Veterinary Medicine, Kasetsart University, 50 Ngamwongwan Road, Bangkok 10900, Thailand; sirikorn.pr@ku.th (S.P.); orawlim826@gmail.com (O.L.); 2 Dermatology Unit, Kasetsart University Veterinary Teaching Hospital, 50 Ngamwongwan Road, Bangkok 10900, Thailand; kritsadasima@gmail.com; 3 Department of Companion Animal Clinical Sciences, Faculty of Veterinary Medicine, Kasetsart University, Bangkok 10900, Thailand; fvetstw@ku.ac.th; 4 Department of Pharmacology, Faculty of Veterinary Medicine, Kasetsart University, 50 Ngamwongwan Road, Bangkok 10900, Thailand; natthasit.t@ku.th (N.T.); fvetpys@ku.ac.th (P.U.)

**Keywords:** *Malassezia pachydermatis*, essential oils, nanoemulsions, antifungal susceptibility, biofilm

## Abstract

*Malassezia pachydermatis* is a yeast pathogen associated with recurrent skin and ear infections in dogs, often complicated by biofilm formation and reduced antifungal susceptibility. We aimed to evaluate the in vitro antifungal activity of essential oils and nanoemulsions of *Zingiber cassumunar* and *Cymbopogon citratus* compared with conventional antifungal agents against planktonic and biofilm forms of *M. pachydermatis*. Preliminary screening of six plant extracts was performed using 12 clinical isolates identified *Z. cassumunar* and *C. citratus* for nanoemulsion formulation. Antifungal susceptibility testing of conventional antifungal agents and nanoemulsions was subsequently conducted using 31 clinical isolates, and nanoemulsions were prepared by high-pressure homogenization. Both essential oils exhibited antifungal activity, and nanoemulsion formulations showed enhanced inhibitory effects compared with the crude oils. Biofilm-associated cells demonstrated reduced susceptibility, particularly to conventional antifungal agents. Terbinafine was the most potent agent against planktonic cells but showed reduced efficacy in biofilms. Nanoemulsions of *Z. cassumunar* and *C. citratus* exhibited improved activity against both forms. These findings suggest that nanoemulsification may enhance the in vitro antifungal performance of essential oils against *M. pachydermatis* biofilms. However, further studies, including mechanistic investigations and in vivo evaluations, are required to confirm their therapeutic potential and safety.

## 1. Introduction

*Malassezia pachydermatis* is a well-known commensal yeast that lives on the skin of dogs and cats with high concentrations of sebaceous glands, for example, on the ear pinna and external ear canal, the ventral neck, the axillary area, the ventral abdomen, the inguinal area, and the interdigital spaces. Dogs and cats with *Malassezia* dermatitis and/or otitis externa usually exhibit chronic, relapsing, and pruritic skin lesions, including erythema, self-induced alopecia, scaling, crusting, and a greasy exudate, with hyperpigmentation and lichenification in the chronic phase. The incidence of opportunistic *Malassezia* dermatitis and otitis externa in otherwise healthy animals is approximately 50% and increases in animals with a breed predisposition or primary hypersensitivity-related diseases [1]. The disease is frequently associated with allergies, ectoparasites, endocrinopathies, environmental humidity, immunomodulatory drug administration, and genetics. This inflammatory skin condition with yeast infestation is primarily a consequence of defects in the skin barrier and underlying diseases of type 1 hypersensitivity [2].

The sustainable treatment of *Malassezia* dermatitis requires identifying and correcting the underlying primary cause, which is challenging. Depending on the severity and distribution of the skin lesions, a common treatment is to administer antifungal drugs systemically and/or topically. Long-term antifungal treatment and the recurrence of *Malassezia* yeast skin infections have led to the emergence of antifungal resistance in *Malassezia* yeasts, particularly to azole drugs, such as miconazole, clotrimazole, itraconazole, and ketoconazole [3]. Concerns regarding antifungal resistance, systemic side effects, and imbalances in the skin microbiota have led to the development of alternative plant- and herbal-based topical products. These products have demonstrated good antimicrobial efficacy in vitro and improved clinical signs in vivo, making them a promising alternative approach to treating *Malassezia* dermatitis and otitis externa [4,5].

However, data are lacking on the in vitro antifungal susceptibility of the planktonic and biofilm forms of *M. pachydermatis*, as well as on the efficacy of plant-derived essential oils against this organism. *Zingiber cassumunar* (*Z. cassumunar*; plai) and *Cymbopogon citratus* (*C. citratus*; lemongrass) are widely used medicinal plants in Thailand and Southeast Asia, traditionally applied for their anti-inflammatory and antimicrobial properties. Previous studies have demonstrated that essential oil derived from *Z. cassumunar* exhibits antimicrobial activity against a broad range of microorganisms, including dermatophytes and yeasts [6], and antifungal activity against *Malassezia furfur* [7]. Similarly, *C. citratus* essential oil, rich in citral, has been widely reported to exhibit strong antifungal activity against various fungal pathogens. Citral has also been shown to exhibit antifungal effects against *M. furfur* and *M. pachydermatis*, supporting its role as a key bioactive component [8,9]. Despite these promising properties, essential oils are inherently hydrophobic and volatile, which may limit their stability and bioavailability in practical applications. Nanoemulsion-based formulations have therefore been proposed to improve the dispersion, stability, and interaction of essential oils with microbial cells. Previous studies have shown that nanoemulsions of essential oils, including *C. citratus*, can enhance antimicrobial activity [10], while nanoemulsions of *Z. cassumunar* have demonstrated favorable physicochemical properties and potential for topical delivery [11]. However, the antifungal activity of these essential oils against biofilm forms of *M. pachydermatis*, as well as the potential of nanoemulsion formulations to enhance their efficacy, remains poorly understood.

In this study, we aimed (1) to determine the antifungal susceptibility of *M. pachydermatis* isolates obtained from dogs with dermatitis in both planktonic and biofilm forms and (2) to evaluate the in vitro antifungal efficacy of commercially available herbal essential oils, with particular emphasis on the essential oil and nanoemulsion formulations of *Z. cassumunar* and *C. citratus* against *M. pachydermatis* in planktonic and biofilm forms.

## 2. Results

### 2.1. Antifungal Susceptibility

We performed in vitro antifungal susceptibility testing of 31 *M. pachydermatis* isolates in both planktonic and biofilm forms. The antifungal susceptibility was quantitatively evaluated using MIC ranges, MIC_50_, and MIC_90_ values. Among all the agents tested, terbinafine was the most potent agent, with the lowest minimum inhibitory concentration (MIC) range (0.03–2 µg/mL), the MIC of the agents at which 50% of the growth of *M. pachydermatis* strains were inhibited (MIC_50_; 0.25 µg/mL), and the MIC of the agents at which 90% of the growth of *M. pachydermatis* strains were inhibited (MIC_90_; 0.5 µg/mL). Among the commonly used antifungal agents, *M. pachydermatis* showed higher susceptibility to ketoconazole than to itraconazole (MIC_90_ of 8 µg/mL vs. 16 µg/mL, respectively). Miconazole, a common component of topical formulations, showed the lowest efficacy, with MIC_50_ and MIC_90_ of 32 µg/mL and >32 µg/mL, respectively.

Under biofilm conditions, *M. pachydermatis* showed reduced susceptibility, with higher MIC and minimum fungicidal concentration (MFC) values for all the tested antifungal agents compared to the planktonic form. Notably, the susceptibility of the biofilm *M. pachydermatis* isolates to terbinafine was markedly reduced, with a 32-fold increase in the MIC range (0.5–16 µg/mL), MIC_50_ (4 µg/mL), and MIC_90_ (16 µg/mL). The ketoconazole and itraconazole MIC values increased 2–4-fold for the biofilm state compared with the planktonic form. The transition to the biofilm state resulted in decreased susceptibility to both agents, with MIC_50_ and MIC_90_ values of 16 µg/mL. Miconazole showed the lowest efficacy against the biofilms, with both MIC_50_ and MIC_90_ > 32 µg/mL. The antifungal susceptibility profiles of *M. pachydermatis* in both planktonic and biofilm forms in this study, compared with those reported previously, are summarized in Table 1.

### 2.2. Essential Oil and Nanoemulsion Susceptibility

The efficacy of the six herbal extracts (*Punica granatum*, *Alpinia galanga*, *Melaleuca alternifolia*, *Z. cassumunar*, *C. citratus*, and *Syzygium aromaticum*) against 12 *M. pachydermatis* isolates was evaluated in both planktonic and biofilm forms, and the MIC ranges, MIC_50_, and MIC_90_ values are summarized in Table 2. All the extracts inhibited the growth of the planktonic form of *M. pachydermatis*, albeit with varying levels of efficacy, and showed consistent fungicidal activity, with MFC values that aligned with the MIC ranges, MIC_50_, and MIC_90_. *C. citratus* was the most potent extract, with MIC/MFC values ranging from 0.078% to 0.312%, followed by the *Z. cassumunar* and *M. alternifolia* extracts with MIC/MFC values ranging from 0.312% to 0.625%. *P. granatum*, *S. aromaticum*, and *A. galanga* were less potent but still effective, with MIC_90_ of 1.25%, 5%, and 2.5%, respectively. When *M. pachydermatis* was grown as a biofilm, the efficacy of all the herbal extracts was reduced, with 4–32-fold higher MIC/MFC values required to inhibit and kill the yeast than for the planktonic form. The most potent extracts against the *M. pachydermatis* biofilms were *C. citratus* and *Z. cassumunar*, with MIC/MFC ranges of 1.25–10%. *P. granatum*, *S. aromaticum*, *A. galanga*, and *M. alternifolia* also required higher concentrations, with MIC/MFC ranges of 2.5–20%, 5–10%, 2.5–20%, and 5–20%, respectively.

**Figure 1:**
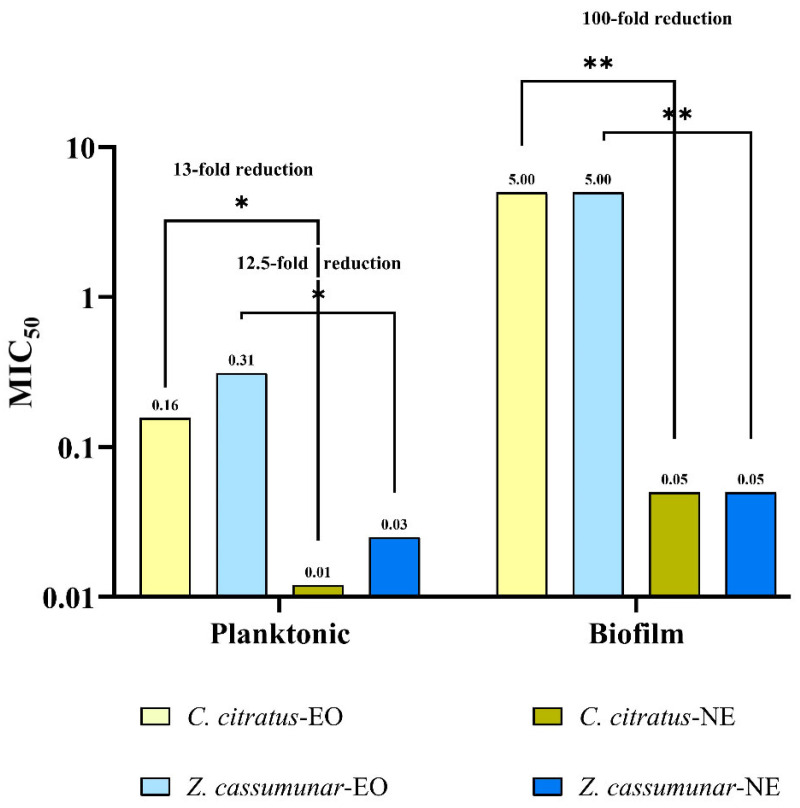
The comparative antifungal efficacy of the pure essential oil and nanoemulsion MIC_50_ values (%) against *M. pachydermatis* isolates in planktonic and biofilm form. The data are presented on a log_10_ scale. Brackets indicate the fold-reduction in MIC between each essential oil and its corresponding nanoemulsion formulation. Asterisks denote statistical significance calculated via unpaired *t*-tests on log_2_ transformed data (* *p* < 0.05, ** *p* < 0.01). EO: essential oil; NE: nanoemulsion.

Among the six extracts tested, *Z. cassumunar* and *C. citratus* were selected for nanoemulsion formulation based on their higher antifungal activity. The antifungal activities of the *Z. cassumunar* and *C. citratus* nanoemulsions against the planktonic and biofilm forms of the 31 *M. pachydermatis* isolates are summarized in Table 2 and Figure 1. The nanoemulsion formulations demonstrated greater potency against the planktonic and biofilm forms of *M. pachydermatis* than their corresponding essential oil extracts, with low MIC/MFC values. Statistical analysis revealed that nanoemulsification significantly enhanced the antifungal efficacy of both the extracts (*p* < 0.05). For the planktonic form of *M. pachydermatis*, the MIC_50_ decreased from 0.156% (essential oil) to 0.012% (nanoemulsion) for *C. citratus* and from 0.312% (essential oil) to 0.025% (nanoemulsion) for *Z. cassumunar*, resulting in 13- and 12.5-fold increases in potency, respectively. Notably, the nanoemulsions exhibited even greater efficacy against biofilms, reducing the MIC_50_ by 100-fold compared with the raw essential oils (*p* < 0.01).

## 3. Discussion

*M. pachydermatis* was the primary isolate in 77.5% of the 40 dogs included in this study, which yielded 31 isolates. This finding is consistent with previous reports that *M. pachydermatis* was isolated from 63% of dogs with otitis externa or dermatitis and from 74.6% of dogs with pruritic disease and otitis [20,21]. The inclusion criteria for skin lesions, namely, yeast infection observed directly under a microscope (as per Section 4) and a cytology score of +2 or higher for *Malassezia* dermatitis/otitis, were therefore highly effective indicators of active infection in dogs and correlated directly with the high primary isolation success rate [22].

The broth microdilution reference method for testing the antifungal susceptibility of yeasts, according to the Clinical and Laboratory Standards Institute (CLSI) M27 guidelines, with modifications [23], is now widely used for antifungal susceptibility testing of *Malassezia* yeasts [23]. However, inconsistencies in antifungal susceptibility have been documented in previous studies owing to the lack of standardized testing protocols, notably, the absence of a uniform culture medium that accounts for *Malassezia*’s lipid-dependent nature, which requires lipid supplementation to grow properly, even for nonlipid-dependent species such as *M. pachydermatis* (see the comparative overview summarized in Table 3). The antifungal susceptibility profiles of the *M. pachydermatis* isolates obtained from dogs with dermatitis/otitis in this study were consistent with those reported for isolates from dogs with dermatitis/otitis and from dogs with normal skin in Japan but differed from those reported in other geographic regions. This may reflect geographical differences that influence antifungal susceptibility. The *M. pachydermatis* isolates from both Thailand and Japan exhibited a similar pattern of low azole sensitivity, including to itraconazole (MIC_50_ = 2 µg/mL, MIC_90_ = 16 µg/mL) and miconazole (MIC_50_ = 32 µg/mL, MIC_90_ > 32 µg/mL); however, they were still susceptible to terbinafine (MIC_50_ = 0.5 µg/mL, MIC_90_ = 2 µg/mL) [14]. Host-related factors, including host species and health status, may play a critical role in the variations in susceptibility. A recent study on *M. pachydermatis* isolates from healthy cats reported greater susceptibility to itraconazole (MIC_50_ = 0.03 µg/mL, MIC_90_ = 2 µg/mL), ketoconazole (MIC_50_ = 0.13 µg/mL, MIC_90_ = 8 µg/mL), miconazole (MIC_50_ = 2 µg/mL, MIC_90_ > 32 µg/mL), and terbinafine (MIC_50_ = 0.13 µg/mL, MIC_90_ = 0.5 µg/mL) [15]. Humans and their pets have close contact, and this proximity carries the risk of zoonotic transmission to immunocompromised people, which they need to be made aware of. Awareness also needs to be raised regarding the increase in low-susceptibility profiles and the emergence of multidrug resistance among *M. pachydermatis* isolates from dogs with and without lesions. Furthermore, research is required to standardize antifungal susceptibility testing and to establish comprehensive epidemiological cutoff values to ensure the accurate interpretation of susceptibility profiles [24].

The results of the antifungal susceptibility test against *M. pachydermatis* in biofilm form in this study were consistent with those of previous studies, which found that the MIC values of all tested antifungal drugs were higher than those for planktonic cells [12,19,25,26]. Although few studies have evaluated the MICs of antifungal agents against biofilm-associated *M. pachydermatis*, terbinafine demonstrated the greatest reduction in antifungal efficacy in our study, with MICs increasing up to 32-fold. In contrast, the azole antifungals, including itraconazole, ketoconazole, and miconazole, exhibited only modest 2–4-fold increases in MICs against biofilm-associated cells. The pronounced reduction in terbinafine efficacy could be attributed to structural and metabolic alterations in the sessile cells within mature biofilms, as well as to differences in the mechanisms of action among antifungal agents. Terbinafine, an allylamine, acts as a fungicidal agent by inhibiting squalene epoxidase, leading to squalene accumulation and cell toxicity, ultimately causing cell death. For the planktonic cell form, terbinafine therefore had the lowest MIC among the azole drugs. However, when microorganisms grow in a biofilm, their metabolic rate decreases. In *M. pachydermatis* biofilms, this leads to slower squalene accumulation in cells and reduced terbinafine sensitivity [27]. The azole group of drugs exerts fungistatic effects by targeting sterol 14-alpha-methylase. This prevents the conversion of lanosterol to ergosterol in the fungal cell membrane, leading to loss of cell membrane function, cell lysis, cell death, and inhibition of fungal growth. Accordingly, the MIC values of the azole drugs were 2–4-fold higher in the biofilm form than in the planktonic form. Notably, even in the planktonic form, the MIC values for itraconazole, ketoconazole, and miconazole were already high in the present study, indicating reduced susceptibility overall. This overall reduced susceptibility, along with the additional increase in MIC in the biofilm form, may be attributed to adaptive changes in sessile cells during biofilm-associated growth, including reduced ergosterol levels in their cell membranes [28]. Azole drugs that target ergosterol biosynthesis are thus less effective in destroying or inhibiting fungal growth than planktonic cells, which have a higher accumulation of ergosterol in their cell membranes and are therefore more susceptible to azole drugs. In this study, we confirmed the effect of biofilm formation on reduced susceptibility or the development of resistance to antifungal drugs in *M. pachydermatis* [12,25]. Antifungal treatment of *Malassezia* yeast skin infections may therefore not be successful unless the fungal biofilm structure is disrupted. An example of this approach is the use of 2% miconazole and 2% chlorhexidine shampoo, which is the first-choice topical treatment of *Malassezia* dermatitis in dogs and cats and is used twice weekly. The synergistic effects of chlorhexidine and miconazole could be explained by chlorhexidine, a cationic biguanide with a positive charge that allows it to bind negatively charged components of the biofilm matrix and the fungal plasma membrane, thereby disrupting the biofilm. Miconazole can then enter and act by inhibiting the growth of the underlying sessile cells [29,30].

In the present study, among the six herbal extracts tested, *C. citratus*, *Z. cassumunar*, and *M. alternifolia* exhibited the highest fungicidal activity against *M. pachydermatis* in planktonic form, consistent with previous studies on the efficacy of these extracts [31,32,33]. However, the findings of different studies on the efficacy of essential oils have been variable for several reasons, including experimental design, test methods, the source and preparation of the essential oil, and strains of *M. pachydermatis* [33]. All the herbal extracts in the present study showed inhibitory activity against the planktonic cells of the *M. pachydermatis* isolated from dogs with dermatitis and did not associate with antifungal susceptibility profiles. These findings are consistent with those of previous studies on herbal extracts that have overcome the antifungal drug resistance in *M. pachydermatis* [34,35,36]. This could be explained by the different mechanisms of synthetic antifungal drugs, which mainly act on a single target or pathway. Herbs and plants have been shown to have multitarget mechanisms for killing or inhibiting fungal growth, including ergosterol biosynthesis, membrane disruption, modulation of energy metabolism via reduced ATPase activity, and the induction of reactive oxygen species, leading to apoptotic oxidative stress [37]. Herbal extracts, such as *S. aromaticum*, are used for their inhibitory effects on the yeast group *Candida albicans*.

Eugenol, a phenylpropanoid, is the major compound in *S. aromaticum* oil, though its proportion varies by plant origin, part, and extraction method [7,8,9]. It has been reported to exhibit antifungal activity against *Candida* spp., potentially through mechanisms such as disruption of cell membrane integrity, alteration of membrane permeability, interference with ATP production and enzyme activity, induction of oxidative stress, and cellular damage, leading to fungal cell death [10]. Several herbs in the Zingiberaceae family, including *A. galanga* and *Z. cassumunar*, have been reported to contain various active compounds such as terpinen-4-ol, α-terpineol, and limonene, which are reported bioactive constituents associated with antimicrobial and antifungal activities [11]. Previous studies have demonstrated that extracts from Zingiberaceae rhizomes can induce morphological alterations in fungal cells, including cell shrinkage, plasmolysis, and leakage of metal ions, suggesting impairment of membrane permeability and cellular homeostasis. These findings support the role of structural and functional damage in the antifungal activity of these plant-derived compounds [38,39]. Moreover, herbal and plant extracts have been reported as multifunctional therapeutic agents, as they display broad-spectrum antimicrobial and antiparasitic actions, anti-inflammatory and healing properties, and antioxidant and antitumor effects [40,41].

We found that *M. pachydermatis* in biofilm form was highly tolerant of all tested essential oils, with MICs increasing 2- to 32-fold compared to planktonic cells. In their study, Aiemsaard et al. [18] demonstrated slightly reduced (2-fold) susceptibility of *M. pachydermatis* biofilms to the *S. aromaticum* essential oil, eugenol, and ketoconazole [18]. To overcome yeast biofilm tolerance to essential oils, the addition of antiseptic compounds, such as chlorhexidine and boric acid, has shown additive and synergistic effects, which increase the efficacy of essential oils in killing yeasts in biofilms [42,43].

Nanoemulsions are among the most promising delivery systems for enhancing the efficacy of antimicrobials and essential oils, particularly against biofilms [44]. Among the six herbal extracts tested, *C. citratus* and *Z. cassumunar* were selected for nanoemulsion formulation in this study due to their high potency against both the planktonic and biofilm forms of *M. pachydermatis*. Biofilm tolerance is largely attributed to the extracellular polymeric substance (EPS) matrix, which serves as a physical and chemical barrier to protect microorganisms. The pronounced antifungal efficacy of the *C. citratus* and *Z. cassumunar* nanoemulsions against these biofilms could be explained by their hydrophobic bioactive constituents, such as citral in *C. citratus* and terpinen-4-ol in *Z. cassumunar*, and by the ability of the nanoemulsions to penetrate biofilms. The encapsulation of essential oils into nanoemulsion systems has been reported to enhance their physicochemical properties, potentially enabling improved dispersion, stability, and interaction with microbial cells. In biofilm-associated systems, nano-sized droplets may facilitate diffusion through the EPS matrix and enhance the delivery of active compounds, leading to membrane disruption, homeostatic disturbance, and ultimately cell death [44,45,46]. However, these mechanisms were not directly investigated in the present study and were inferred from previous reports. Our findings, confirming that *C. citratus* and *Z. cassumunar* nanoemulsions exhibit significantly increased potency against both planktonic and biofilm forms of *M. pachydermatis*, demonstrate that a nanodelivery system is a highly promising approach for developing natural anti-*Malassezia* products for the treatment of *Malassezia* dermatitis. These results align with those of previous studies that have integrated essential oil nanoemulsions into biomedical materials and therapeutic products, such as wound dressings, hydrogels, and topical creams, as well as into the surface coatings of medical devices [47,48,49].

Despite these promising findings, this study has several limitations. First, the essential oils were commercially obtained and characterized by manufacturer-issued certificates of analysis; no independent chemical profiling (e.g., GC–MS or LC–MS) was performed. Second, physicochemical properties of the nanoemulsion formulations—droplet size distribution, polydispersity index, zeta potential, and stability—were not characterized after preparation, which may affect their antifungal performance. Third, this study focused on in vitro antifungal activity, and the underlying molecular mechanisms or interactions between active compounds and fungal cells were not investigated. In addition, comparisons with standard topical antiseptics commonly used in veterinary dermatology (e.g., chlorhexidine or boric acid) [50] and evaluation of potential synergistic interactions were not included. Cytotoxicity and irritation assessments were also not performed. Therefore, the safety, tolerability, and clinical applicability of these formulations remain to be determined. Further studies should include comprehensive chemical and physicochemical characterization, mechanistic investigations, comparative efficacy studies with standard topical agents, evaluation of synergistic effects, and in vivo safety assessments to better establish their clinical potential.

## 4. Materials and Methods

### 4.1. Ethical Statement and Sample Collection

The study protocol was reviewed and approved by the Institutional Animal Care and Use Committee of Kasetsart University (approval no. ACKU64-VET-041; approval date: 9 August 2021), and the sample collection from the animals was performed at the Dermatology Unit of the Kasetsart University Veterinary Teaching Hospital, Bang Khen Campus, Thailand. Forty dogs presenting with dermatitis and demonstrating a yeast infection, as observed directly under a microscope (Olympus Corporation, Tokyo, Japan), with a positive yeast cytology score of +2 or higher in line with the semiquantitative grading system described by Nascente et al. [22], were enrolled for skin sample collection. The veterinarian used sterile cotton swabs moistened with normal saline and swabbed the skin at the lesion site, such as the ear pinna, neck, interdigital area, groin, or inguinal area.

### 4.2. Yeast Isolation and Identification

**Figure 2:**
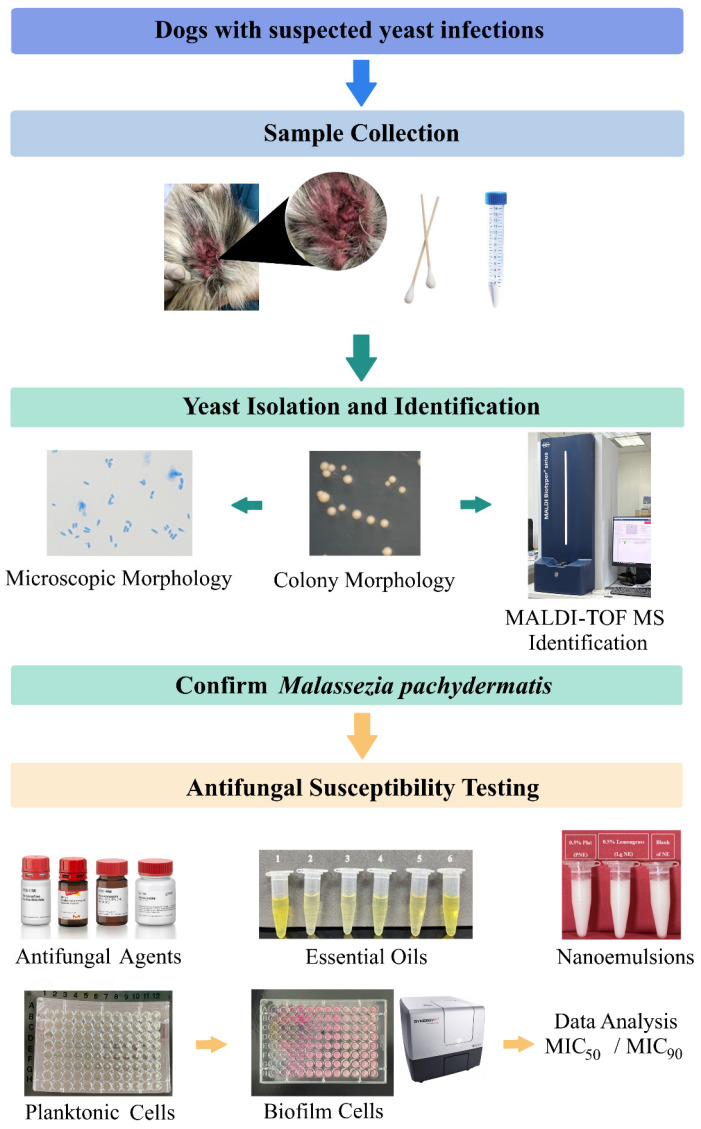
Schematic overview of the study design. Skin swabs from dogs with suspected yeast infections were collected. Yeast isolation and identification were performed by culture, morphological characterization, and MALDI-TOF MS to confirm *Malassezia pachydermatis*. Antifungal susceptibility of planktonic and biofilm forms was evaluated for conventional antifungal agents, essential oils, and nanoemulsions based on MIC ranges, MIC_50_, and MIC_90_.

The skin swabs were inoculated on Sabouraud dextrose agar (SDA; Difco, Sparks, MD, USA) containing oxytetracycline (50 mg/L; Oxycline, Bangkok, Thailand) [51]. All the inocula were incubated at 30 °C and examined daily for colony growth for 14 days. The yeast colonies were examined for growth rate, colony appearance, microscopic morphology, and urea hydrolysis. *Malassezia* yeast-like colonies were subcultured on SDA and subjected to species identification by MALDI-TOF MS (Bruker Daltonics, Bremen, Germany). The *M. pachydermatis* strains were stored at −80 °C in Sabouraud dextrose broth (SDB; Difco, Sparks, MD, USA) with 30% glycerol for use as a culture collection in further experiments. The overall study workflow is illustrated in Figure 2.

### 4.3. Selection of Essential Oils and Preparation of Nanoemulsions and Antifungal Agents

All the herbal extracts in the form of essential oils, namely, *Punica granatum*, *Z. cassumunar*, *Alpinia galanga*, *Melaleuca alternifolia*, *Syzygium aromaticum*, and *C. citratus*, were commercially obtained with a certificate of analysis from SNP Co., Ltd., Muang Chonburi, Thailand, and TCFF Industry Co., Ltd., Phra Nakorn Si Ayutthaya, Thailand. The parts used and the chemical components of the tested essential oils are listed in Table 3. Based on preliminary antifungal screening against 12 isolates of *M. pachydermatis*, the essential oils of *Z. cassumunar* and *C. citratus* were selected for nanoemulsion formulation due to their comparatively higher antifungal activity and lower MIC values in both planktonic and biofilm forms.

The preparation of the nanoemulsions for *Z. cassumunar* and *C. citratus* followed an internally developed protocol designed for herbal oil formulations. This process produced an oil-in-water nanoemulsion using high-pressure homogenization (15,000 psi, five cycles), which resulted in oil droplets with diameters ranging from 150 to 200 nm. The nanoemulsion was formulated by combining an aqueous phase with an oil phase: 0.5% herbal essential oil, mixed surfactant, propylene glycol, paraben, sodium ethylenediaminetetraacetic acid (EDTA), and purified water.

Ketoconazole, itraconazole, miconazole, and terbinafine were selected for susceptibility testing as representative antifungal agents commonly used in veterinary practice for the treatment of *Malassezia* infections, covering both topical and systemic therapies [50]. These agents were included as reference drugs to establish baseline antifungal susceptibility and to provide a comparative context for interpreting the activity of the tested essential oils and nanoemulsion formulations. All antifungal agents were purchased from Sigma-Aldrich (St. Louis, MO, USA). Stock solutions of the antifungal agents were dissolved in dimethyl sulfoxide (DMSO; Sigma-Aldrich, St. Louis, MO, USA).

### 4.4. Antifungal Susceptibility Testing in Planktonic Form

**Figure 3:**
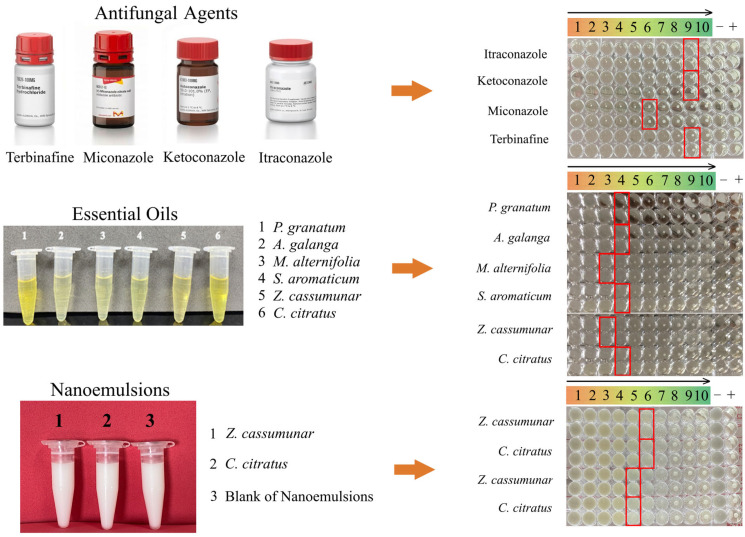
Representative images of test agents and broth microdilution assays for planktonic antifungal susceptibility testing. Wells 1–10 represent two-fold serial dilutions from highest to lowest concentrations tested (see Table 4). “−” indicates the negative control, and “+” indicates the growth control. Red boxes indicate MIC endpoints.

Antifungal susceptibility testing was performed using the broth microdilution method based on the CLSI document M27–A3 [52], with specific modifications for *Malassezia* spp. [23]. To prepare test solutions, stock solutions of water-insoluble antifungal agents were dissolved in DMSO and then diluted in SDB supplemented with 1% Tween 80 (SDB–T_80_; Sigma-Aldrich, St. Louis, MO, USA). Essential oils and nanoemulsion formulations, obtained as liquid preparations, were first diluted in distilled water, and the final concentrations were adjusted with SDB–T_80_. All working solutions were prepared in separate tubes and serially diluted two-fold in SDB–T_80_ to obtain 10 concentrations for each substance, as detailed in Table 4. A volume of 100 µL of each test agent concentration was dispensed into 96-well round-bottom microdilution plates (Corning Costar, Corning, NY, USA). Tween 80 served as an emulsifying agent to facilitate the dispersion of hydrophobic compounds. *M. pachydermatis* was grown on SDA for 72 h at 35 °C before being adjusted to an optical density of 0.425–0.435 at 530 nm using spectrophotometry and diluted in SDB–T_80_ for a final concentration of 1–5 × 10^4^ CFU/mL. Antifungal susceptibility was determined by visually reading the MICs after incubation at 35 °C for 72 h [53]. Wells were scored for turbidity using a standardized scale (0–4) relative to the drug-free growth control. For antifungal agents included in this study, the MIC was defined as the lowest concentration yielding a score of 2 (approximately 50% reduction in growth), whereas for essential oils and nanoemulsion formulations, the MIC was the lowest concentration yielding a score of 0 (optically clear). MFCs were determined by subculturing from MIC wells into fresh SDB–T_80_, with the MFC defined as the lowest concentration showing no fungal growth. *Candida parapsilosis* ATCC 22019 was used as a quality control strain. The experimental setup and visual determination of MIC endpoints are shown in Figure 3.

### 4.5. Biofilm Formation and Antifungal Testing

*M. pachydermatis* inocula were prepared as described for planktonic susceptibility test to achieve a final concentration of 1–5 × 10^4^ CFU/mL. The cell suspensions were dispersed into 96-well flat-bottom microdilution plates (Corning Costar, Corning, NY, USA) and incubated at 35 °C for 72 h to allow biofilm formation [17]. The supernatant in each well was then gently removed by pipetting, and test agents (antifungal agents, essential oils, and nanoemulsions) were added, followed by incubation at 35 °C for 72 h. Cell viability within the biofilms was assessed using alamarBlue solution (Bio-Rad, Hercules, CA, USA), which was added to each well and incubated at 35 °C for an additional 3 h. Antifungal susceptibility was determined by measuring absorbance at 570 nm using a microplate reader (Agilent Technologies, Santa Clara, CA, USA) [18]. The MICs were determined as the lowest concentration that resulted in a 50% reduction in absorbance relative to the positive control. MFCs were determined as described for planktonic susceptibility testing.

### 4.6. Data Analysis and Efficacy Comparison

Antifungal susceptibility to antifungal agents, essential oils, and nanoemulsions was analyzed for the 31 *M. pachydermatis* isolates obtained from dogs with dermatitis, in planktonic and biofilm form, and reported as MIC ranges, MIC_50_, MIC_90_, and MFC ranges, calculated using WHONET (version 20.11.5). For a comparative statistical analysis, the raw MIC values were log_2_-transformed to achieve normality. The efficacy of the essential oils versus the corresponding nanoemulsion formulations of the *C. citratus* and *Z. cassumunar* extracts was evaluated by comparing the MIC_50_ values for the planktonic and biofilm growth forms using unpaired *t*-tests. All the statistical analyses and graphical representations were performed using GraphPad Prism version 10.6.1 (GraphPad Software, San Diego, CA, USA), and *p* < 0.05 was considered statistically significant.

## 5. Conclusions

In this study, *M. pachydermatis* isolates demonstrated reduced in vitro susceptibility to conventional azoles. While terbinafine remained susceptible to the planktonic form, it showed less activity against biofilms. Of all essential oils tested, *C. citratus* and *Z. cassumunar* had the highest efficacy, especially when formulated as nanoemulsions, which enhance antifungal activity against biofilms. These findings suggest that nanoemulsion-based delivery systems may improve in vitro antifungal performance of essential oils against *M. pachydermatis*. However, as this study was limited to in vitro assays, further studies are required before clinical application, including mechanisms, comparison with standard topical agents, assessment of potential synergistic interactions, and evaluation of cytotoxicity, tolerability, and in vivo safety.

## Figures and Tables

**Table 1:** Comparison of the in vitro susceptibility profiles of planktonic and biofilm *M. pachydermatis* isolates from dogs with dermatitis in the present study and those from previously reported data.

Antifungal Agent	Test Condition	Test Isolate (N)	MIC Range	MIC_50_	MIC_90_	Reference
Itraconazole	Planktonic	31	0.5–16	8	16	This study
	Biofilm	31	2–32	16	32	This study
	Planktonic	52	0.125–2	0.25	0.5	[12]
	Biofilm	52	4–16	16	16	[12]
	Planktonic	82	0.01–0.25	0.06	0.125	[13]
	Planktonic	29	0.125–>32	2	16	[14]
	Planktonic	27	<0.06–16	0.03	2	[15]
	Planktonic	25	0.03–8	0.25	4	[16]
	Planktonic	40	0.01–0.5	0.01	0.25	[17]
Ketoconazole	Planktonic	31	0.5–8	4	8	This study
	Biofilm	31	4–32	16	32	This study
	Planktonic	17	-	0.019	-	[18]
	Biofilm	6	-	0.038	-	[18]
	Planktonic	10	-	0.03	-	[19]
	Biofilm	10	-	>16	-	[19]
	Planktonic	40	0.01–0.5	0.06	0.5	[17]
	Planktonic	25	0.25–>16	2	8	[16]
	Planktonic	82	0.01–0.25	0.06	0.125	[13]
	Planktonic	27	<0.06–>32	0.13	8	[15]
Miconazole	Planktonic	31	2–>32	32	>32	This study
	Biofilm	31	16–>32	>32	>32	This study
	Planktonic	29	8–>32	32	>32	[14]
	Planktonic	27	<0.06–>32	2	>32	[15]
	Planktonic	40	0.01–0.5	0.25	0.5	[17]
Terbinafine	Planktonic	31	0.031–2	0.25	0.5	This study
	Biofilm	31	0.5–16	16	16	This study
	Planktonic	25	0.03–0.25	0.125	0.25	[16]
	Planktonic	29	0.125–2	0.5	2	[14]
	Planktonic	27	<0.02–8	0.13	0.5	[15]

The MIC_50_ and MIC_90_ values were defined as the minimum concentrations of antifungal agents required to inhibit the growth of 50% and 90% of the *M. pachydermatis* isolates, respectively.

**Table 2:** The efficacy of six herbal extracts against 12 *Malassezia pachydermatis* isolates in planktonic and biofilm form and two nanoemulsions against the planktonic and biofilm forms of 31 *M. pachydermatis* isolates.

Herbal Extract	Condition	MIC Range	MIC_50_	MIC_90_	Mode
*Z. cassumunar*	Planktonic	0.312–0.625	0.312	0.625	0.625
	Biofilm	1.25–10	5	10	5
*P. granatum*	Planktonic	0.312–1.25	0.312	0.625	0.312
	Biofilm	2.5–20	10	20	10
*S. aromaticum*	Planktonic	0.312–5	0.312	5	0.312
	Biofilm	5–10	10	10	10
*A. galanga*	Planktonic	0.625–2.5	0.625	2.5	0.625
	Biofilm	2.5–20	5	10	5
*M. alternifolia*	Planktonic	0.312–0.625	0.312	0.312	0.312
	Biofilm	5–20	10	20	10
*C. citratus*	Planktonic	0.078–0.312	0.156	0.312	0.156
	Biofilm	1.25–10	5	10	5
*Z. cassumunar* nanoemulsion	Planktonic	0.012–0.05	0.025	0.05	0.025
	Biofilm	0.025–0.1	0.05	0.1	0.05
*C. citratus* nanoemulsion	Planktonic	0.006–0.025	0.012	0.025	0.012
	Biofilm	0.025–0.05	0.05	0.05	0.05

The MIC_50_ and MIC_90_ values were defined as the minimum concentrations of the herbal extract at which 50% and 90% of the growth of *M. pachydermatis* strains were inhibited, respectively. Mode is the MIC that represents the most frequently obtained MIC.

**Table 3:** Parts used and chemical components of the herbs used for testing.

Testing Substance	Botanical Name	Part Used	Chemical Composition	Purchased from
Pomegranate extract liquid	*Punica granatum* L.	Fruit peel obtained by Soxhlet extraction	-Phenolic compounds, including anthocyanin compounds, delphinidin-3-glucoside, and 5,5-diglucoside -Ellagitannins and ellagic acid	SNP Co., Ltd., Thailand
Galanga extract liquid	*Alpinia* *galanga* L.	Rhizome obtained by steam distillation	-Phenolic compounds, including (+)-catechin, quercetin, catechol, isorhamnetin, and gallic, and trans-cinnamic and protocatechuic acids -Flavonoid and antioxidant	TCFF Industry Co., Ltd., Thailand
Tea tree extract liquid	*Melaleuca alternifolia* L.	Leaves obtained by steam distillation	-Terpinen-4-ol, γ-terpinene, p-cymene, α-terpinene, and terpineol	TCFF Industry Co., Ltd. Thailand
Plai extract liquid	*Zingiber cassumunar* Roxb.	Rhizome obtained by hydro distillation	-Compound D, D-acetate, DMPBD, sabinene, γ-terpinene, and terpinen-4-ol	TCFF Industry Co., Ltd. Thailand
Lemongrass extract liquid	*Cymbopogon citratus* (DC.) *Stapf*	Leaves obtained by steam distillation	-Geranial and neral from citral -Myrcene and limonene -Antioxidant	TCFF Industry Co., Ltd. Thailand
Clove extract liquid	*Syzygium aromaticum* (L.)	Flower buds obtained by steam distillation	-Phenolic compounds, including eugenol -Cyperene, phenethyl isovalerate, and cis-thujopsene	TCFF Industry Co., Ltd. Thailand

Note: DMPBD, (E)-1-(3,4-methoxyphenyl) butadiene.

**Table 4:** Solvents and concentration ranges of antifungal agents, essential oils, and nanoemulsion formulations.

Test Agent Type	Agent	Solvent	Concentration Range
Antifungal agent	Itraconazole	DMSO	32–0.0625 (µg/mL)
Antifungal agent	Ketoconazole	DMSO	32–0.0625 (µg/mL)
Antifungal agent	Miconazole	DMSO	32–0.0625 (µg/mL)
Antifungal agent	Terbinafine	DMSO	8–0.0156 (µg/mL)
Essential oil	*Z. cassumunar*	DW	40–0.078 (%)
Essential oil	*P. granatum*	DW	40–0.078 (%)
Essential oil	*A. galanga*	DW	20–0.039 (%)
Essential oil	*M. alternifolia*	DW	20–0.039 (%)
Essential oil	*S. aromaticum*	DW	10–0.019 (%)
Essential oil	*C. citratus*	DW	10–0.019 (%)
Nanoemulsion	*Z. cassumunar*	DW	40–0.078 (%)
Nanoemulsion	*C. citratus*	DW	10–0.019 (%)

Note: DMSO, dimethyl sulfoxide; DW, distilled water.

## Data Availability

The data presented in this study are available within the article.
